# Potential to Improve Therapy of Chronic Myeloid Leukemia (CML), Especially for Patients with Older Age: Incidence, Mortality, and Survival Rates of Patients with CML in Switzerland from 1995 to 2017

**DOI:** 10.3390/cancers13246269

**Published:** 2021-12-14

**Authors:** Michael Daskalakis, Anita Feller, Jasmine Noetzli, Nicolas Bonadies, Volker Arndt, Gabriela Maria Baerlocher, The NICER Working Group

**Affiliations:** 1 Department of Hematology, Inselspital, Bern University Hospital, University of Bern, Freiburgstrasse 18, 3010 Bern, Switzerland; info@cabinetnoetzli.ch (J.N.); nicolas.bonadies@insel.ch (N.B.); 2 Department of BioMedicalResearch (DMBR), University of Bern, Murtenstrasse 40, 3008 Bern, Switzerland; 3 Foundation National Institute for Cancer Epidemiology and Registration (NICER), University of Zurich, Seilergraben 49, 8001 Zurich, Switzerland; anita.feller@nicer.org (A.F.); v.arndt@dkfz-heidelberg.de (V.A.); 4 National Agency for Cancer Registration (NACR) Operated by NICER, University of Zurich, 8001 Zurich, Switzerland; 5 Cabinet Noetzli, Avenue de Rumine 35, 1005 Lausanne, Switzerland; 6 Unit of Cancer Survivorship, Division of Clinical Epidemiology and Aging Research, German Cancer Research Center, 69120 Heidelberg, Germany

**Keywords:** CML, incidence, mortality, relative survival, tyrosine kinase inhibitor

## Abstract

**Simple Summary:**

In a population-based study of chronic myeloid leukemia (CML) patients in Switzerland, we confirmed an increase in relative survival for all age groups over the last decades. This was primarily based on the stable age-adjusted rate of incidence and a substantial decrease of the age-adjusted mortality rate. Investigating data from four different study periods, before and after introduction of tyrosine kinase inhibitors and their more potent second- and third-generation compounds, we found higher increases in relative survival for older patients at later time periods compared to younger CML patients. However, for the last study period (2013–2017), the five-year relative survival (RS) in the elderly population reached only 53% compared to 89% in younger patients, implicating additional potential to improve CML therapy, especially in the elderly population.

**Abstract:**

Background: Tyrosine kinase inhibitors (TKI) substantially improved chronic myeloid leukemia (CML) prognosis. We aimed to describe time period- and age-dependent outcomes by reporting real-world data of CML patients from Switzerland. Methods: Population-based incidence, mortality, and survival were assessed for four different study periods and age groups on the basis of aggregated data from Swiss Cantonal Cancer Registries. Results: A total of 1552 new CML cases were reported from 1995 to 2017. The age-standardized rate (ASR) for the incidence remained stable, while the ASR for mortality decreased by 50–80%, resulting in a five-year RS from 36% to 74% over all four age groups. Importantly, for patients <60 years (yrs), the five-year RS increased only in earlier time periods up to 92%, whereas for older patients (+80 yrs), the five-year RS continued to increase later, however, reaching only 53% until 2017. Conclusions: This is the first population-based study of CML patients in Switzerland confirming similar data compared to other population-based registries in Europe. The RS increased significantly in all age groups over the last decades after the establishment of TKI therapy. Interestingly, we found a more prominent increase in RS of patients with older age at later observation periods (45%) compared to patients at younger age (10%), implicating a greater benefit from TKI treatment for elderly occurring with delay since the establishment of TKI therapy. Our findings suggest more potential to improve CML therapy, especially for older patients.

## 1. Introduction

Chronic myeloid leukemia (CML) is a clonal myeloproliferative neoplasia defined by the translocation t(9;22). Annual incidence rates are reported with 0.6 to 2.0 cases per 100,000 person-years (py), accounting for approximately 10–15% of newly diagnosed cases of adult leukemia [1]. Geographic, ethnic, and age variations might contribute to the variability of incidence among different registries, and predisposing factors for the disease are not well understood. Large population-based studies on CML in the western world have been set up in the USA [2] and Europe [3], in the latter case mainly in the United Kingdom [4], the Netherlands [5], Sweden [6,7], and France [8]. Lower incidence rates are reported in Asian countries [9], where CML seems to affect a younger age group, and more patients belong to the high and intermediate Sokal risk groups [10]. An increased incidence of CML was found among Japanese atomic bomb survivors [11]. An association of CML incidence to lifestyle, dietary factors, and a higher risk of disease progression associated with tobacco use were described [12,13]. Occupational pesticide exposure has been considered as a potential risk factor; however, no strong association with CML has been established [14]. The median age at diagnosis of CML ranges from 57 to 60 years (yrs), with slightly more males than females (ratio 1.2–1.7) affected [15,16]. Most patients diagnosed with CML are asymptomatic and are often diagnosed in an early chronic stage during a routine physical examination or blood testing. Only a smaller proportion of cases present with accelerated phase (AP) or blast crisis (blast phase, BP) at diagnosis [17].

In the past, CML was associated with a poor prognosis and short time of survival; treatment options were limited to arsenic containing compounds [18] and single splenic irradiation in the 19th and early 20th centuries, respectively. Introduction of busulfan [19] in the 1960s and hydroxyurea in the 1970s reduced leukocytosis and clinical symptoms. Usage of interferon alpha (INF α) for the first-time achieved cytogenetic remissions in the 1980s [20,21,22,23,24,25]. Since the 1990s, the introduction of an allogeneic stem cell transplantation in patients <50 yrs of age resulted in a 5 year RS rate of up to 74% [26], being improved by the adoptive immunotherapy of donor lymphocyte infusion (DLI) in the treatment of CML recurrence [27,28]. At the beginning of the 21st century, the introduction of BCR-ABL1 targeted therapy with tyrosine kinase inhibitors (TKI) rapidly improved the prognosis, with overall survival rate improving from 20% to 80–90% [29].

Over the last 20 yrs after the introduction of imatinib, the first-generation TKI, in 2001 in Switzerland, more potent TKI such as nilotinib, dasatinib, and bosutinib, the second-generation TKI, and ponatinib, the third-generation TKI, were available from 2005 (first line from 2007) and 2010, respectively. Due to the variation of the five available TKIs in terms of their potency, activity against kinases other than BCR-ABL1, as well as activity against ABL1 mutations, pharmacokinetics, and adverse effect profiles, the armamentarium to treat and monitor patients with CML has allowed for a better personalization of the therapy.

Whereas in chronic phase of CML, the single treatment with TKI is standard of care, the outcome of blast phase CML remains poor, with no consensus frontline treatment approach. The use of hypomethylating agents such as decitabine might improve the treatment of CML as previously demonstrated for myelodysplastic diseases such as chronic myelomonocytic leukemia (CMML) [30]. Retrospective analysis has shown that combined front-line treatment with a second- or third-generation TKI and a hypomethylating agent or intensive chemotherapy leads to improved response rates and outcome in myeloid blastic crisis followed by allogeneic stem cell transplantation [31].

Due to the effectiveness of TKI treatment, disease-monitoring strategies have also changed over time, using peripheral blood (PB) instead of bone marrow (BM) for the molecular monitoring and aiming for earlier and deeper molecular responses. CML management has been summarized in several CML treatment guidelines [32,33,34,35]. With optimal TKI response, life expectancy is approaching that of the general population and will result in an extended increase of CML prevalence [36]. Several studies have shown that TKI treatment is effective and well tolerated in all age groups. Therefore, age-related biological differences do not seem to affect the prognosis of CML in the elderly [37,38,39,40].

Here, we describe for the first time the real-world demographic characteristics, annual cases, incidence, mortality, and relative survival of CML patients from population-based cancer registries in Switzerland and compared them with other European and non-European studies. Trends were compared for 1995–2000, 2001–2006, 2007–2012, and 2013–2017, representing four time periods before the introduction of TKI treatment and after the establishment of first-, second-, and third-generation TKI therapy.

## 2. Materials and Methods

### 2.1. Study Design

We performed a population-based study using data from the Swiss Cantonal Cancer Registries (CCRs) and aggregated by the National Agency for Cancer Registration (NACR) operated by the National Institute for Cancer Epidemiology and Registration (NICER). Results were compared to reports from other population-based CML registries/cohorts. Cases reported to the CCRs in Switzerland from 1995 to 2017 were included. Data were stratified for the following four different time periods: the era before the introduction of TKI treatment (1995–2000), after the establishment of the first-generation TKI therapy (2001–2006), the era after the introduction of second-generation TKIs (2007–2012), and the period after the use of third-generation TKI therapy (2013–2017).

### 2.2. Data Sources and Inclusion Criteria

CML cases reported to the National Agency for Cancer Registration between 1995 and 2017 were included. The NACR is collecting and harmonizing data from CCRs and provides cancer registration data for the entirety of Switzerland. All cancer cases were coded according to the third revision of the International Classification of Diseases for Oncology (ICD-O-3). Cancer cases diagnosed prior to the introduction of ICD-O-3 were recorded from ICD-O-2 to ICD-O-3 by the CCRs. Survival status information was collected by passive (linkage with federal mortality data) and active follow up (verification of vital status with the cantonal registration offices). A detailed description of the organization of cancer registration in Switzerland and its data collection procedures can be found elsewhere [41,42]. Due to the gradual introduction of cantonal cancer registration, national population coverage for this study increased from 57.1% in 1995–2000 to 88.5% in 2013–2017.

Incident CML cases were identified by the following ICD-O-3 codes: 9863/3, 9875/3, 9876/3. Overall, 95.2% of all cases were morphologically verified. The proportion of death certificate-only (DCO) cases was 2.3%, indicating a high completeness of case ascertainment.

Mortality data; mid-year population estimates; and cantonal death rates by age, sex, and calendar year were obtained from the Swiss Federal Statistical Office (SFSO), referring to all persons with permanent residence status in Switzerland. The mortality information is based on civil registries and on SFSO standardized death certificates, indicating the cause of death. The coding of death certificates and the selection of the underlying cause of death is carried out by the SFSO using the 10th revision of the International Statistical Classification of Diseases and Health-Related Problems (ICD-10) [43], following international standards.

### 2.3. Analytic Methods

We calculated five-year age-specific, crude, and age-standardized incidence and mortality rates with corresponding 95% confidence intervals (95%CI) for the time periods 1995–2000, 2001–2006, 2007–2012, and 2013–2017. Age-standardized rates (ASR) were calculated using the direct method and the European standard population 1976 [44] as a reference. Case frequencies for the whole of Switzerland were obtained by applying the observed incidence rates (by age, sex, and period) of the cantons covered by cancer registration to the cantons without registration, assuming homogeneity between cantons with and without cancer registration. Relative survival (RS) was estimated by dividing the observed survival after diagnosis by the survival as expected in the general population of corresponding sex, age, calendar year, and region of residence. Observed survival (OS) was estimated on the basis of transformation of the cumulative hazards [45]. Expected survival was estimated using the Ederer II method [45]. We calculated OS and RS up to 5-yrs after diagnosis for the time periods listed above using period analysis [45]. Age-stratified analyses were performed for the age categories <60 yrs, 60–69 yrs, 70–79 yrs, and 80+ yrs. RS estimates for all age groups combined were age-standardized using weights (standard 1) from the International Cancer Survival Standards [46]. Significance tests for RS were applied according to the method described by Parkin and Hakulinen [47] comparing five-year RS between time periods.

Statistical analyses were performed using Stata/MP version 15.1 (StataCorp LLC, College Station, TX, USA).

## 3. Results

### 3.1. Study Population

From 1995 to 2017, a total of 1552 newly diagnosed patients with CML were registered in the Swiss CCRs (Table 1): 323 cases between 1995 and 2000, 385 cases between 2001 and 2006, 390 cases between 2007 and 2012, and 454 cases between 2013 and 2017. Age-stratified analyses were performed for the age categories <60, 60–69, 70–79, and 80+ yrs. In our population, male CML patients in comparison to female were predominant, with 1.35 with respect to the complete observation time (Table 1). Median age at diagnosis ranged from 59.5 to 67 yrs in the different time periods, and for all patients (“total”), most patients were in the age group <60 yrs. In total, the distribution of all patients in the four different age categories was quite stable over the complete observation time. It must be pointed out that the percentage of population covered by cancer registration in Switzerland increased over the complete observation time (57.1% in 1995–2000, 58.8% in 2001–2006, 66.6% in 2007–2012, and 88.5% in 2013–2017).

### 3.2. Incidence

**Figure 1:**
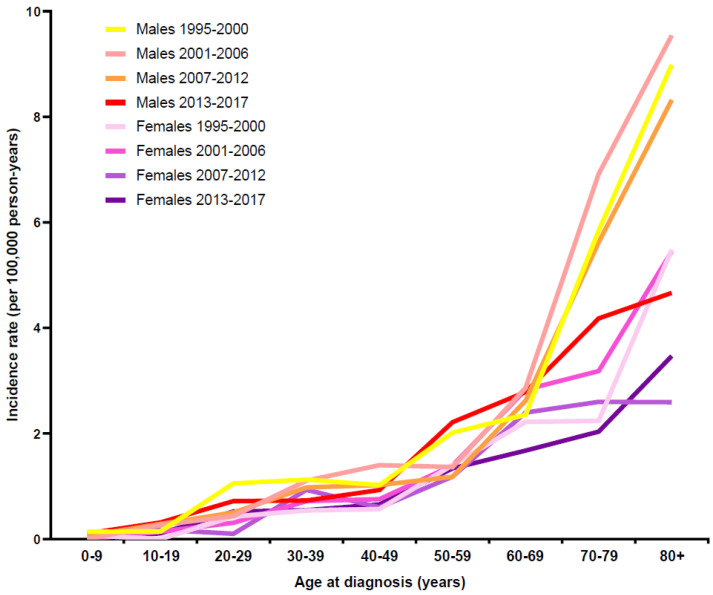
Age-specific incidence of CML patients in Swiss CCRs by sex and time period.

Figure 1 depicts the increase of the age-specific incidence in CML in both sex groups. Interestingly, the incidence of CML for male versus female patients increased more after the age of 60 yrs for the first three time periods. For all patients, the crude incidence rate (CIR) was the lowest at higher ages for the last time period (2013–2017). Nevertheless, for this time period, the CIR was still higher for males than females in patients above the age of 50 yrs.

As seen in Table S1 the annual cases over the different time periods were similar. The ASR for incidence remained stable over the first and second time periods (1.10 and 1.17 for all patients (“total”), respectively) and decreased slightly for the third (2007–2012) and fourth time period (2013–2017) to 0.99 per 100,000 py (Table S1). Differentiated for the sex groups, the ASR of males and females decreased slightly over the complete observation period from 1.44 to 1.23 and from 0.82 to 0.75 per 100,000 py, respectively (Table S1).

### 3.3. Mortality

**Figure 2:**
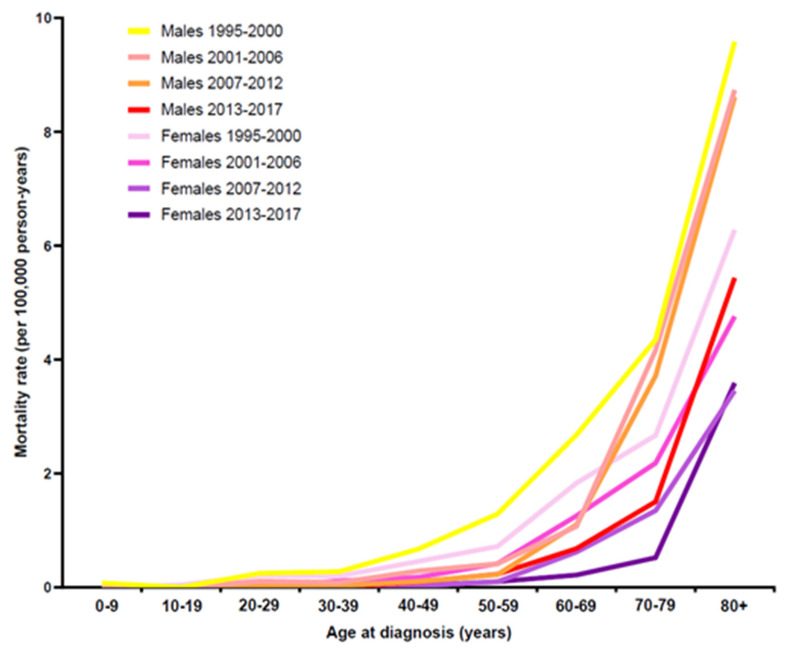
Mortality of CML patients in Swiss CCRs by sex and time period.

As shown in Figure 2, age-specific mortality-rates of CML patients increased generally with higher age in both sex groups. The age-specific mortality-rate, however, decreased with later time periods. Comparing the same time periods, we found that males demonstrated higher mortality rates than females. For all patients, the age-specific mortality rate was lowest for the latest time period.

In males, a clear decrease of age-specific mortality rates was observed above the age of 40 yrs for the later three time periods in comparison to the first time period. The age-specific male mortality rate of the second (2001–2006) and third (2007–2012) time periods approximated above the age of 70 yrs with the male patient curve of the first time period. For the females, the age-specific mortality rate curves decreased with each time period, except for the age group of 80+ yrs.

The ASR for mortality of CML patients in the Swiss CCRs impressively decreased for all patients over the complete observation period, from 0.80 for 1995 to 2000 to 0.21 per 100,000 py for 2013 to 2017 (Table S2). The decrease of ASR in mortality was slightly more prominent in male patients compared to females. In the male cohort, ASR decreased from 1.00 (1995 to 2000) to 0.30 per 100,000 py (2013 to 2017), whereas in the female cohort, ASR diminished from 0.64 (1995 to 2000) to 0.13 per 100,000 py (2013 to 2017) (Table S2).

### 3.4. Survival

**Figure 3:**
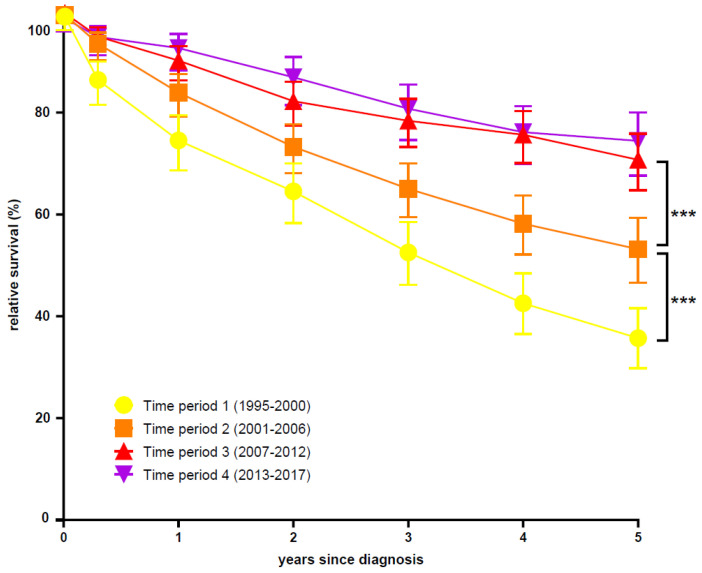
Age-standardized relative survival of CML patients in Swiss CCRs by time period. Significant statistical analysis is shown as follows: ***, <0.001; (see also Tables S3 and S4).

The age-standardized RS of Swiss CML patients steadily decreased with years after diagnosis, with continuous improvements over the four study time periods. For the first time period, the age-standardized RS was 35.7% at 5 yrs after diagnosis of CML, whereas for the third and fourth time periods, it reached 70.7% and 74.4%, respectively. Importantly, for these patients the age-standardized five-year RS curve clearly increased over the time periods 1 to 3. No additional increase, however, could be observed in time period 4 (Figure 3).

**Figure 4:**
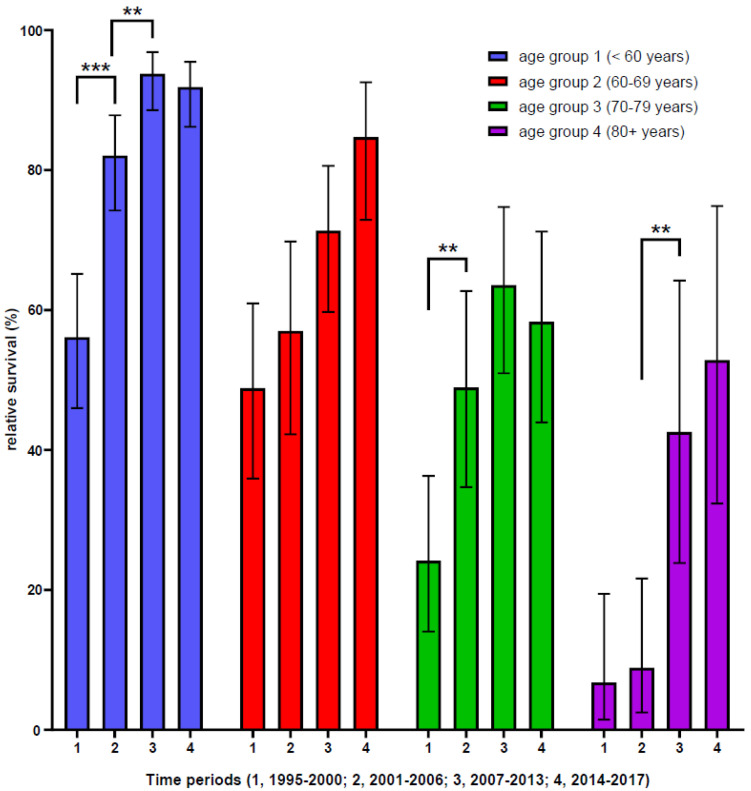
Relative five-year survival of CML patients in Swiss CCRs grouped by age and time periods. Significance of five-year relative survival of CML patients is shown as follows: **, <0.01; ***, <0.001; (see also Tables S3 and S4).

We performed an additional stratified analysis of the five-year RS in dissecting the four different age groups in different time periods. The five-year RS only increased up to the third time period, but not for the fourth time period for CML patients below the age of 60 yrs. In contrast, the five-year RS, especially for CML patients above the age of 80 yrs, increased substantially from the third time period onward (Figure 4).

A significant improvement in five-year OS and RS over the complete observed time period was found for all patients and all age groups. Taking all age groups together, we found that the RS of CML patients significantly increased at 5 yrs from 35.7% for 1995 to 2000, to 53.2% for 2001 to 2006, to 70.7% for 2007 to 2012, and up to 74.4% for 2013 to 2017 (see Table S3). Detailed age-related RS curves over 5 yrs after diagnosis are depicted in Figure S1A–D.

In Table S5, we summarize reports from other population-based CML studies. In most of the studies, there was also a significant increase in the five-year RS over the last decades from primarily 20–40% up to 80–90% in the recent years.

## 4. Discussion

This is the first report of real-world population-based data from CML patients diagnosed in Switzerland between 1995 and 2017. We compared time trends of demographic characteristics, annual cases, incidences, mortalities, and survival for four different age-groups (<60 yrs, 60–69 yrs, 70–79 yrs, 80+ yrs) at four relevant time periods. These included before the introduction of TKI treatment (1995–2000), and after the establishment of first-generation TKI therapy (2001–2006), within the era of first- and second-generation TKI therapy (2007–2012), and the period after establishment of the second-generation and the access to third-generation TKI therapy (2013–2017).

In our Swiss CML population, the median age at diagnosis for all patients ranged from 59.5 to 67 yrs over the complete observation period, which is in a similar range as reported by the U.S. SEER database (median age, 65 yrs) [2] and the Lithuanian national hematological disease monitoring system (HESS; median age, 62 yrs) [48]. Other CML registries reported lower median age at diagnosis (35 to 55 yrs), the lowest being described in the Asian populations of Hong Kong and Singapore [9], as well as from India [49]. Geographic, socio-economic, and ethnic variations might contribute to these variabilities, and thus far, potential etiologic or predisposing factors cannot be excluded.

In accordance with other registries and cohorts, the incidence and mortality of CML in the Swiss population increased by age, showing a peak at around 80 to 85 yrs of age. In addition, CML was found to be more common in males than females. For our cohort, the CIRs (for all patients between 1.24 and 1.48 per 100,000 py over the complete observation period), the ASRs for the incidence (for all patients 0.99 to 1.17 per 100,000 py from 1995 to 2017) as well as for the mortality (for all patients decreasing from 0.80 to 0.21% over the complete time period) were within the range reported from other European CML population-based registries [3]. Higher CIRs have been described from the SEER database (CIR, 1.75); however, a different reference population was used for the calculation. In contrast, lower CIRs ranging from 0.58 to 0.75 per 100,000 py have been reported from registries of Russia (2009–2012) [50], India (1976–2005) [49], and Calgary (2011–2015) [51]. Furthermore, several reports suggest a lower CML incidence in some Asian countries (e.g., Taiwan Cancer Registry (1997–2007), Singapore Cancer Registry (1998–2002), Thailand, or China) [9,52,53], an observation that was also described within the U.S. SEER database comparing the Asian U.S. population to Caucasians [2]. As discussed in two very recent publications, differences in ASR for incidence and mortality seem to be influenced by geographical and socio-economic factors [54,55]. In addition, methodological factors of the different studies can also not be excluded [7].

The considerable improvement of the age-standardized RS in our Swiss cohort of CML patients over the last 20 years is based on the fairly stable incidence and the substantial decrease in mortality. This is in line with reports from other population-based registries [2,3]. During the first observation period (1995–2000) of the Swiss cohort, the CML therapy consisted mostly of chemotherapeutic agents, interferon alpha [56], and allogeneic hematopoietic stem cell transplantation (HSCT) for patients <60 yrs of age with good performance status. Not unexpectedly, this first observation period showed the lowest RS. Due to the introduction of the targeted therapy with BCR-ABL1 TKIs in 2001, the ongoing developments in molecular diagnostics and its resulting refinements in diagnostics, as well as in accordance with the change in aging populations, the outcome of CML has changed substantially in the last decades. Over 90% of CML patients who were diagnosed in chronic phase (CP) and treated with a TKI demonstrated an excellent OS [57,58,59] and a nearly normal life expectancy compared to the general population [36,60]. As a result of the prolonged survival with TKI treatment, prevalence of CML has risen steadily within the last decade and is projected to increase continuously for the coming years [8].

Importantly, in delineating the RS for the different age groups and time periods, we found important benefits associated with the changes in CML management for all age groups over the last 20 years. The benefit of higher generation TKI treatments seems to reach a plateau of RS in younger CML patients, whereas there seems to be an ongoing improvement of RS in older CML patients. The delayed effect of increased RS in the older patient population might be due to a certain caution of physicians to prescribe TKI treatment to frail patients or patients with many comorbidities. Of note, comorbidities do not have an impact on TKI treatment but a negative effect on OS, indicating that comorbidities, rather than CML itself, determine survival of CML patients, as has been reported in a previous study [61]. TKI treatment should therefore be equally considered for all CML patients, independent of age.

**Figure 5:**
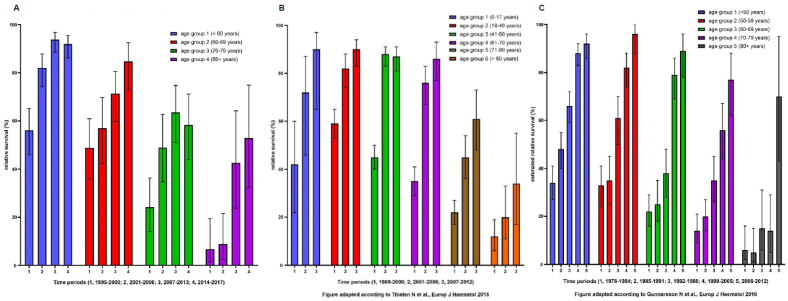
Relative five-year survival grouped by age and time periods of CML patients in Swiss CCRs (**A**), of CML patients from the Dutch population-based registry (**B**), and of CML patients from the Swedish population-based registry (**C**).

Due to different time periods, age, and potentially sex distribution in other population-based CML registries, comparison of RS needs to account for generally confounding factors and may be interpreted with caution. Taking the five-year RS data until approximately 2000 and before the introduction of TKI therapy, comparable results between the different population-based registries can be found (Figure 5 and Table S5).

The Swiss data depict a five-year RS of 36%, which is in line with the U.S. SEER and Dutch databases reporting 36%, as well as the Lithuanian study group describing 33%. Interestingly, the report of five French registries from Penot et al. [62] described a five-year RS of 64% for the time period of 1987–1999. For French patients, a possible explanation might be the centralized and rigid adherence to treatment centers of excellence within registries and study groups. When we compared the five-year RS after the introduction of second-generation first-line TKI treatment (2007–2013), our own data showed a RS of 71% over all age groups, which was similar to the Dutch study group (79%) [5]. An even better five-year RS was reported by the U.K. (90%) [4,63] and Swedish (80%) study groups [6,64], whereas the five-year RS in the Lithuanian study group [48] had not been reached at the time of publication. Again, early integration of TKIs in CML therapy recommendations and guidelines, as well as the mandatory treatment at selected centers of excellence for elderly CML patients might be an explanation for better treatment results and RS in these countries.

## 5. Conclusions

In conclusion, our population-based CCR data of CML patients diagnosed in Switzerland between 1995 and 2017 was found to be similar for distribution of age, sex, CIR, and ASR of incidence and mortality compared to other population-based registries in Europe. The RS dramatically increased after the establishment of TKI therapy, which corroborates the observations from all other study groups. Importantly, the RS was clearly higher for patients with younger ages and improved over the last decades for all age groups. Most interestingly, we found in our cohort of CML patients that the benefit from CML management during the last time periods including higher generation TKI seemed to impact the RS of patients with older age more substantially than that of patients at a younger age. To improve the overall RS for CML patients with the current therapeutic options, age-independent wider access to and use of, as well as more consistent and patient-tailored adherence to TKI treatment should be propagated. Due to the lack of population-based systematic collection of information regarding types of treatment, side effects, responses, and comorbidities, interpretation of the available data has its limitations. With the new law on National Cancer Registration that has been enforced in Switzerland in 2020, reporting of incident cancer cases has become mandatory with additional information on first-line treatment and responses, and this will allow us more detailed analysis in future years.

## Figures and Tables

**Table 1:** Distribution of CML cases reported to Swiss cancer registries for 1995–2000, 2001–2006, 2007–2012 and 2013–2017.

Variation	1995–2000			2001–2006			2007–2012				2013–2017	
	*n*	% *	MedianAge (IQR)	*n*	% *	Median Age (IQR)	*n*	% *	Median Age (IQR)	*n*	% *	Median Age (IQR)
Males	**186**	**57.6**	**59.5 (41–73)**	**210**	**54.6**	**64.5 (46–76)**	**227**	**58.2**	**66 (47–77)**	**268**	**59.0**	**61 (48.5–72)**
<60 years	93	50.0		85	40.5		88	38.8		129	48.1	
60–69 years	25	13.4		35	16.7		42	18.5		54	20.2	
70–79 years	40	21.5		54	25.7		55	24.2		54	20.2	
80+ years	28	15.1		36	17.1		42	18.5		31	11.6	
Females	**137**	**42.4**	**65 (50–80)**	**175**	**45.5**	**67 (53–79)**	**163**	**41.8**	**64 (47–76)**	**186**	**41.0**	**63 (50–78)**
<60 years	51	37.2		61	34.9		65	39.9		81	43.6	
60–69 years	27	19.7		38	21.7		41	25.2		34	18.3	
70–79 years	22	16.1		34	19.4		32	19.6		31	16.7	
80+ years	37	27.0		42	24.0		25	15.3		40	21.5	
Total	**323**	**100.0**	**63 (45–77)**	**385**	**100.0**	**66 (49–77)**	**390**	**100.0**	**65 (47–77)**	**454**	**100.0**	**61.5 (49–74)**
<60 years	144	44.6		146	37.9		153	39.2		210	46.3	
60–69 years	52	16.1		73	19.0		83	21.3		88	19.4	
70–79 years	62	19.2		88	22.9		87	22.3		85	18.7	
80+ years	65	20.1		78	20.3		67	17.2		71	15.6	

IQR: Interquartile range. Population covered by cancer registration: 57.1% in 1995–2000, 58.8% in 2001–2006, 66.6% in 2007–2012 and 88.5% in 2013–2017. *, Percentage of bolded numbers of males and females add up to 100% for both sexes, and non-bolded percentages of each age group add up to the total of 100%.

## Data Availability

Data available on reasonable request from the authors.

## Supplementary Materials

The following are available online at https://www.mdpi.com/article/10.3390/cancers13246269/s1, Figure S1: Relative survival time of CML patients in Swiss CCRs grouped by age and time periods: (**A**) Relative survival time of CML patients in Swiss CCR of time period 1 (1995–2000); (**B**) Relative survival time of CML patients in Swiss CCR of time period 2 (2001–2006); (**C**) Relative survival time of CML patients in Swiss CCR of time period 3 (2007–2012); (**D**) Relative survival time of CML patients in Swiss CCR of time period 4 (2013–2017), Table S1: Incidence of CML, 1995–2000, 2001–2006, 2007–2012, and 2013–2017, Table S2: Mortality of CML, 1995–2000, 2001–2006, 2007–2012, and 2013–2017, Table S3: OS and RS at 5 yrs of CML patients in the Swiss CCR, Table S4: Significance of five-year RS of CML patients in the Swiss CCR, Table S5: Summary of other population-based CML studies.

## Abbreviations

ABL1	Abelson murine leukemia gene 1
ASR	age-standardized rates per 100,000 person years
BCR	breakpoint cluster region
CCR	cantonal cancer registry
CI	confidence interval
CML	chronic myeloid leukemia
CP-CML	chronic phase of CML
CIR	crude incidence rate
CR	crude rates per 100,000 person years
INF α	interferon alpha
IQR	interquartile range
NACR	National Agency for Cancer Registration
OS	overall survival
RS	relative survival
SFSO	Swiss Federal Statistical Office
Phi	Philadelphia chromosome
py	person years
t(9;22)	translocation chromosome 9 and 22
TKI	tyrosine kinase inhibitor
yrs	years

## References

[1] Siegel R.L., Miller K.D., Jemal A. (2017). Cancer Statistics, 2017. CA A Cancer J. Clin..

[2] Chen Y., Wang H., Kantarjian H., Cortes J. (2013). Trends in chronic myeloid leukemia incidence and survival in the United States from 1975 to 2009. Leuk. Lymphoma.

[3] Hoffmann V.S., Baccarani M., Hasford J., Lindoerfer D., Burgstaller S., Sertic D., Costeas P., Mayer J., Indrak K., Everaus H. (2015). The EUTOS population-based registry: Incidence and clinical characteristics of 2904 CML patients in 20 European Countries. Leukemia.

[4] Roman E., Smith A., Appleton S., Crouch S., Kelly R., Kinsey S., Cargo C., Patmore R. (2016). Myeloid malignancies in the real-world: Occurrence, progression and survival in the UK’s population-based Haematological Malignancy Research Network 2004-15. Cancer Epidemiol..

[5] Thielen N., Visser O., Ossenkoppele G., Janssen J. (2016). Chronic myeloid leukemia in the Netherlands: A population-based study on incidence, treatment, and survival in 3585 patients from 1989 to 2012. Eur. J. Haematol..

[6] Bjorkholm M., Ohm L., Eloranta S., Derolf A., Hultcrantz M., Sjoberg J., Andersson T., Hoglund M., Richter J., Landgren O. (2011). Success story of targeted therapy in chronic myeloid leukemia: A population-based study of patients diagnosed in Sweden from 1973 to 2008. J. Clin. Oncol. Off. J. Am. Soc. Clin. Oncol..

[7] Hoglund M., Sandin F., Simonsson B. (2015). Epidemiology of chronic myeloid leukaemia: An update. Ann. Hematol..

[8] Delord M., Foulon S., Cayuela J.M., Rousselot P., Bonastre J. (2018). The rising prevalence of chronic myeloid leukemia in France. Leuk. Res..

[9] Au W.Y., Caguioa P.B., Chuah C., Hsu S.C., Jootar S., Kim D.W., Kweon I.Y., O’Neil W.M., Saikia T.K., Wang J. (2009). Chronic myeloid leukemia in Asia. Int. J. Hematol..

[10] Jootar S. (2012). CML treatment in Asia-Pacific region. Hematology.

[11] Hsu W.L., Preston D.L., Soda M., Sugiyama H., Funamoto S., Kodama K., Kimura A., Kamada N., Dohy H., Tomonaga M. (2013). The incidence of leukemia, lymphoma and multiple myeloma among atomic bomb survivors: 1950–2001. Radiat. Res..

[12] Kabat G.C., Wu J.W., Moore S.C., Morton L.M., Park Y., Hollenbeck A.R., Rohan T.E. (2013). Lifestyle and dietary factors in relation to risk of chronic myeloid leukemia in the NIH-AARP Diet and Health Study. Cancer Epidemiol. Biomark. Prev..

[13] Lauseker M., Hasford J., Saussele S., Kremers S., Kraemer D., Lindemann W., Hehlmann R., Pfirrmann M. (2017). Smokers with chronic myeloid leukemia are at a higher risk of disease progression and premature death. Cancer.

[14] Van Maele-Fabry G., Duhayon S., Lison D. (2007). A systematic review of myeloid leukemias and occupational pesticide exposure. Cancer Causes Control CCC.

[15] Apperley J.F. (2015). Chronic myeloid leukaemia. Lancet.

[16] Jabbour E., Kantarjian H. (2020). Chronic myeloid leukemia: 2020 update on diagnosis, therapy and monitoring. Am. J. Hematol..

[17] Cortes J.E., Talpaz M., O’Brien S., Faderl S., Garcia-Manero G., Ferrajoli A., Verstovsek S., Rios M.B., Shan J., Kantarjian H.M. (2006). Staging of chronic myeloid leukemia in the imatinib era: An evaluation of the World Health Organization proposal. Cancer.

[18] Conan-Doyle A. (1882). Notes on a case of leucocythaemia. Lancet.

[19] (1968). Chronic granulocytic leukaemia: Comparison of radiotherapy and busulphan therapy. Report of the Medical Research Council’s working party for therapeutic trials in leukaemia. Br. Med. J..

[20] Talpaz M., Kantarjian H.M., McCredie K., Trujillo J.M., Keating M.J., Gutterman J.U. (1986). Hematologic remission and cytogenetic improvement induced by recombinant human interferon alpha A in chronic myelogenous leukemia. N. Engl. J. Med..

[21] Hehlmann R., Heimpel H., Hasford J., Kolb H.J., Pralle H., Hossfeld D.K., Queisser W., Loffler H., Hochhaus A., Heinze B. (1994). Randomized comparison of interferon-alpha with busulfan and hydroxyurea in chronic myelogenous leukemia. The German CML Study Group. Blood.

[22] Bonifazi F., de Vivo A., Rosti G., Guilhot F., Guilhot J., Trabacchi E., Hehlmann R., Hochhaus A., Shepherd P.C., Steegmann J.L. (2001). Chronic myeloid leukemia and interferon-alpha: A study of complete cytogenetic responders. Blood.

[23] Goldman J.M., Baughan A.S., McCarthy D.M., Worsley A.M., Hows J.M., Gordon-Smith E.C., Catovsky D., Batchelor J.R., Goolden A.W., Galton D.A. (1982). Marrow transplantation for patients in the chronic phase of chronic granulocytic leukaemia. Lancet.

[24] Pavlu J., Szydlo R.M., Goldman J.M., Apperley J.F. (2011). Three decades of transplantation for chronic myeloid leukemia: What have we learned?. Blood.

[25] Silver R.T., Woolf S.H., Hehlmann R., Appelbaum F.R., Anderson J., Bennett C., Goldman J.M., Guilhot F., Kantarjian H.M., Lichtin A.E. (1999). An evidence-based analysis of the effect of busulfan, hydroxyurea, interferon, and allogeneic bone marrow transplantation in treating the chronic phase of chronic myeloid leukemia: Developed for the American Society of Hematology. Blood.

[26] Hansen J.A., Gooley T.A., Martin P.J., Appelbaum F., Chauncey T.R., Clift R.A., Petersdorf E.W., Radich J., Sanders J.E., Storb R.F. (1998). Bone marrow transplants from unrelated donors for patients with chronic myeloid leukemia. N. Engl. J. Med..

[27] Dazzi F., Szydlo R.M., Cross N.C., Craddock C., Kaeda J., Kanfer E., Cwynarski K., Olavarria E., Yong A., Apperley J.F. (2000). Durability of responses following donor lymphocyte infusions for patients who relapse after allogeneic stem cell transplantation for chronic myeloid leukemia. Blood.

[28] Raiola A.M., Van Lint M.T., Valbonesi M., Lamparelli T., Gualandi F., Occhini D., Bregante S., di Grazia C., Dominietto A., Soracco M. (2003). Factors predicting response and graft-versus-host disease after donor lymphocyte infusions: A study on 593 infusions. Bone Marrow Transplant..

[29] Kantarjian H., O’Brien S., Jabbour E., Garcia-Manero G., Quintas-Cardama A., Shan J., Rios M.B., Ravandi F., Faderl S., Kadia T. (2012). Improved survival in chronic myeloid leukemia since the introduction of imatinib therapy: A single-institution historical experience. Blood.

[30] Stomper J., Rotondo J.C., Greve G., Lübbert M. (2021). Hypomethylating agents (HMA) for the treatment of acute myeloid leukemia and myelodysplastic syndromes: Mechanisms of resistance and novel HMA-based therapies. Leukemia.

[31] Saxena K., Jabbour E., Issa G., Sasaki K., Ravandi F., Maiti A., Daver N., Kadia T., DiNardo C.D., Konopleva M. (2021). Impact of frontline treatment approach on outcomes of myeloid blast phase CML. J. Hematol. Oncol..

[32] Radich J.P., Deininger M., Abboud C.N., Altman J.K., Berman E., Bhatia R., Bhatnagar B., Curtin P., DeAngelo D.J., Gotlib J. (2018). Chronic Myeloid Leukemia, Version 1.2019, NCCN Clinical Practice Guidelines in Oncology. J. Natl. Compr. Cancer Netw. JNCCN.

[33] Hochhaus A., Baccarani M., Silver R.T., Schiffer C., Apperley J.F., Cervantes F., Clark R.E., Cortes J.E., Deininger M.W., Guilhot F. (2020). European LeukemiaNet 2020 recommendations for treating chronic myeloid leukemia. Leukemia.

[34] Baccarani M., Deininger M.W., Rosti G., Hochhaus A., Soverini S., Apperley J.F., Cervantes F., Clark R.E., Cortes J.E., Guilhot F. (2013). European LeukemiaNet recommendations for the management of chronic myeloid leukemia: 2013. Blood.

[35] Hochhaus A., Saussele S., Rosti G., Mahon F.X., Janssen J., Hjorth-Hansen H., Richter J., Buske C. (2017). Chronic myeloid leukaemia: ESMO Clinical Practice Guidelines for diagnosis, treatment and follow-up. Ann. Oncol. Off. J. Eur. Soc. Med. Oncol..

[36] Bower H., Bjorkholm M., Dickman P.W., Hoglund M., Lambert P.C., Andersson T.M. (2016). Life Expectancy of Patients With Chronic Myeloid Leukemia Approaches the Life Expectancy of the General Population. J. Clin. Oncol. Off. J. Am. Soc. Clin. Oncol..

[37] Crugnola M., Castagnetti F., Breccia M., Ferrero D., Trawinska M.M., Abruzzese E., Annunziata M., Stagno F., Tiribelli M., Binotto G. (2019). Outcome of very elderly chronic myeloid leukaemia patients treated with imatinib frontline. Ann. Hematol..

[38] Lauseker M., Gerlach R., Worseg W., Haferlach T., Tauscher M., Hasford J., Hoffmann V.S. (2019). Differences in treatment and monitoring of chronic myeloid leukemia with regard to age, but not sex: Results from a population-based study. Eur. J. Haematol..

[39] Seo H.Y., Ko T.H., Hyun S.Y., Song H., Lim S.T., Shim K.Y., Lee J.I., Kong J.H. (2019). Tyrosine Kinase Inhibitor Dosing Patterns in Elderly Patients With Chronic Myeloid Leukemia. Clin. Lymphoma Myeloma Leuk..

[40] Lokesh K.N., Pehalajani J.K., Loknatha D., Jacob L.A., Babu M.C.S., Rudresha A.H., Rajeev L.K., Smitha S.C., Ashok K.P., Madhumathi D.S. (2020). CML in Elderly: Does Age Matter?. Indian J. Hematol. Blood Transfus. Off. J. Indian Soc. Hematol. Blood Transfus..

[41] Arndt V., NICER Working Group (2016). Population-based cancer registration and research in Switzerland: Examples, limitations and perspectives. Swiss Cancer Bull..

[42] Noseda G. (2018). The future of cancer registration in Switzerland. Swiss Cancer Bull.

[43] Classification of Diseases (ICD-10). https://www.who.int/classifications/icd/icdonlineversions/en/.

[44] Waterhouse J., Muir C., Correa P., Powell J. (1976). Cancer Incidence in Five Continents.

[45] Dickman P.W., Coviello E., Hills M. (2015). Estimating and modeling relative survival. Stata J..

[46] Corazziari I., Quinn M., Capocaccia R. (2004). Standard cancer patient population for age standardising survival ratios. Eur. J. Cancer.

[47] Parkin D., Hakulinen T. (1991). Analysis of Survival. Cancer Registration: Principles and Methods.

[48] Beinortas T., Tavoriene I., Zvirblis T., Gerbutavicius R., Jurgutis M., Griskevicius L. (2016). Chronic myeloid leukemia incidence, survival and accessibility of tyrosine kinase inhibitors: A report from population-based Lithuanian haematological disease registry 2000-2013. BMC Cancer.

[49] Dikshit R.P., Nagrani R., Yeole B., Koyande S., Banawali S. (2011). Changing trends of chronic myeloid leukemia in greater Mumbai, India over a period of 30 years. Indian J. Med. Paediatr. Oncol..

[50] Kulikov S.M., Vinogradova O., Chelysheva E., Tishchenko I.A., Galaiko M.A., Lazareva O.V., Senderova O.M., Pepeliaeva V.M., Meresii S.V., Luchinin A.S. (2014). Incidence of chronic myeloid leukemia in 6 regions of Russia according to the data of the 2009-2012 population-based study. Ter. Arkhiv..

[51] Nguyen L.T., Guo M., Naugler C., Rashid-Kolvear F. (2018). Incidence of chronic myeloid leukemia in Calgary, Alberta, Canada. BMC Res. Notes.

[52] Kim D.W., Banavali S.D., Bunworasate U., Goh Y.T., Ganly P., Huang H., Irving I., Jootar S., Goh H.G., Koh L.P. (2010). Chronic myeloid leukemia in the Asia-Pacific region: Current practice, challenges and opportunities in the targeted therapy era. Leuk. Res..

[53] Chang C.S., Lee K., Yang Y.H., Lin M.T., Hsu C.N. (2011). Estimation of CML incidence: Disagreement between national cancer registry and health claims data system in Taiwan. Leuk. Res..

[54] Ning L., Hu C., Lu P., Que Y., Zhu X., Li D. (2020). Trends in disease burden of chronic myeloid leukemia at the global, regional, and national levels: A population-based epidemiologic study. Exp. Hematol. Oncol..

[55] Lin Q., Mao L., Shao L., Zhu L., Han Q., Zhu H., Jin J., You L. (2020). Global, Regional, and National Burden of Chronic Myeloid Leukemia, 1990-2017: A Systematic Analysis for the Global Burden of Disease Study 2017. Front. Oncol..

[56] Kantarjian H.M., O’Brien S., Cortes J.E., Shan J., Giles F.J., Rios M.B., Faderl S.H., Wierda W.G., Ferrajoli A., Verstovsek S. (2003). Complete cytogenetic and molecular responses to interferon-alpha-based therapy for chronic myelogenous leukemia are associated with excellent long-term prognosis. Cancer.

[57] Hoglund M., Sandin F., Hellstrom K., Bjoreman M., Bjorkholm M., Brune M., Dreimane A., Ekblom M., Lehmann S., Ljungman P. (2013). Tyrosine kinase inhibitor usage, treatment outcome, and prognostic scores in CML: Report from the population-based Swedish CML registry. Blood.

[58] Hoffmann V.S., Baccarani M., Hasford J., Castagnetti F., Di Raimondo F., Casado L.F., Turkina A., Zackova D., Ossenkoppele G., Zaritskey A. (2017). Treatment and outcome of 2904 CML patients from the EUTOS population-based registry. Leukemia.

[59] Hehlmann R., Lauseker M., Saussele S., Pfirrmann M., Krause S., Kolb H.J., Neubauer A., Hossfeld D.K., Nerl C., Gratwohl A. (2017). Assessment of imatinib as first-line treatment of chronic myeloid leukemia: 10-year survival results of the randomized CML study IV and impact of non-CML determinants. Leukemia.

[60] Sasaki K., Strom S.S., O’Brien S., Jabbour E., Ravandi F., Konopleva M., Borthakur G., Pemmaraju N., Daver N., Jain P. (2015). Relative survival in patients with chronic-phase chronic myeloid leukaemia in the tyrosine-kinase inhibitor era: Analysis of patient data from six prospective clinical trials. Lancet Haematol..

[61] Saussele S., Krauss M.P., Hehlmann R., Lauseker M., Proetel U., Kalmanti L., Hanfstein B., Fabarius A., Kraemer D., Berdel W.E. (2015). Impact of comorbidities on overall survival in patients with chronic myeloid leukemia: Results of the randomized CML study IV. Blood.

[62] Penot A., Preux P.M., Le Guyader S., Collignon A., Herry A., Dufour V., Monnereau A., Woronoff A.S., Troussard X., Pons E. (2015). Incidence of chronic myeloid leukemia and patient survival: Results of five French population-based cancer registries 1980-2009. Leuk. Lymphoma.

[63] Smith A.G., Painter D., Howell D.A., Evans P., Smith G., Patmore R., Jack A., Roman E. (2014). Determinants of survival in patients with chronic myeloid leukaemia treated in the new era of oral therapy: Findings from a UK population-based patient cohort. BMJ Open.

[64] Gunnarsson N., Sandin F., Hoglund M., Stenke L., Bjorkholm M., Lambe M., Olsson-Stromberg U., Richter J., Sjalander A. (2016). Population-based assessment of chronic myeloid leukemia in Sweden: Striking increase in survival and prevalence. Eur. J. Haematol..

