# Supplementary material for: Potential to Improve Therapy of Chronic Myeloid Leukemia (CML), Especially for Patients with Older Age: Incidence, Mortality, and Survival Rates of Patients with CML in Switzerland from 1995 to 2017
Source: Cancers (Basel). 2021 Dec 14;13(24):6269. doi: 10.3390/cancers13246269 (PMC8699480; doi:10.3390/cancers13246269)
Supplement: Supplementary file 1 [file cancers-13-06269-s001.zip › cancers-1474330-supplementary.pdf]

Supplementary materials: Potential to Improve Therapy of Chronic Myeloid Leukemia (CML), Especially for Patients with Older Age: Incidence, Mortality, and Survival Rates of Patients with CML in Switzerland from 1995 to 2017

Michael Daskalakis, Anita Feller, Jasmine Noetzli, Nicolas Bonadies, Volker Arndt, Gabriela Maria Baerlocher and the NICER Working Group

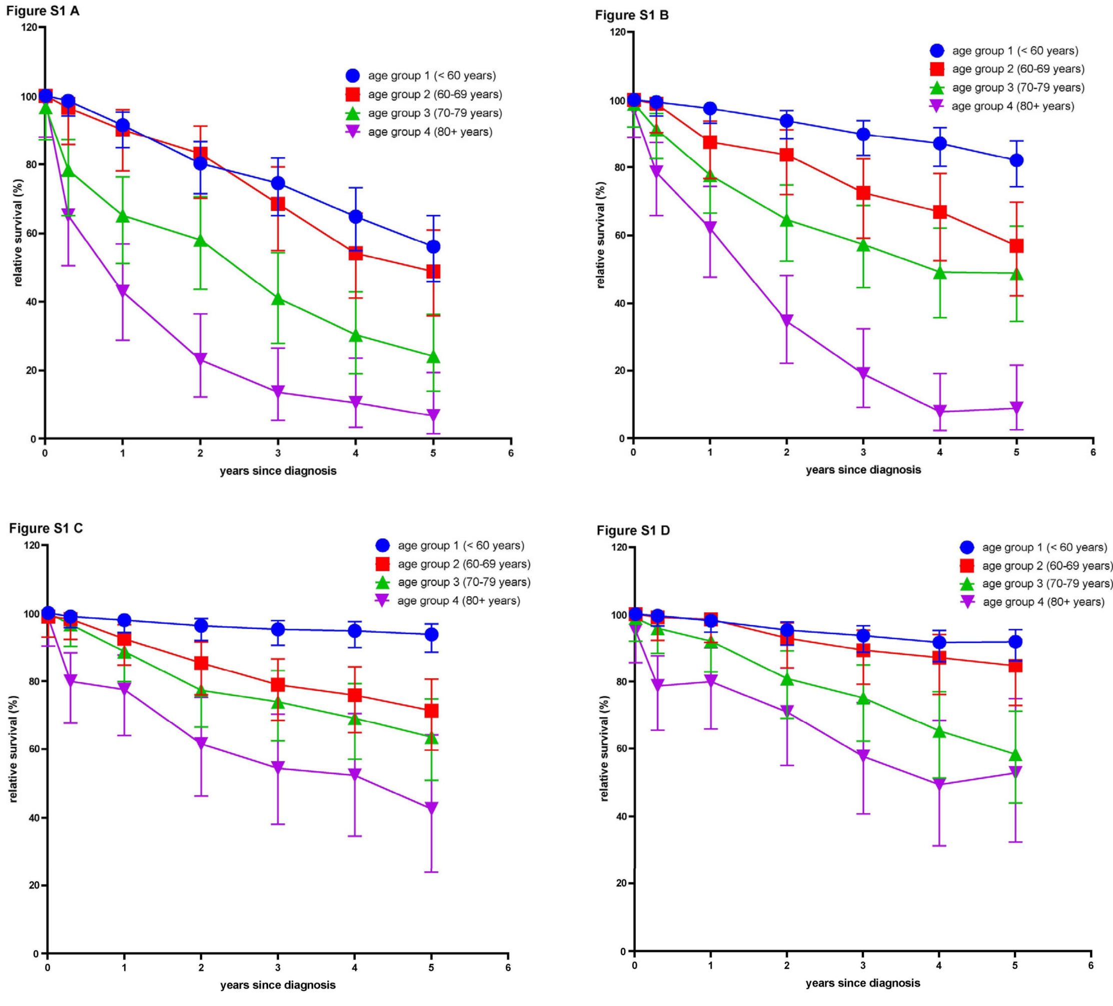

Figure S1. Relative survival time of CML patients in Swiss CCRs grouped by age and time periods: (**A**), relative survival time of CML patients in Swiss CCR of time period 1 (1995–2000); (**B**), relative survival time of CML patients in Swiss CCR of time period 2 (2001–2006); (**C**), relative survival time of CML patients in Swiss CCR of time period 3 (2007–2012); (**D**), relative survival time of CML patients in Swiss CCR of time period 4 (2013–2017).

Table S1: Incidence of CML, 1995–2000, 2001–2006, 2007–2012, and 2013–2017.

Variation	1995–2000			2001–2006			2007–2012			2013–2017		
	**Annual Cases***	**CIR**	**ASR (95% CI)**	**Annual Cases***	**CIR**	**ASR (95% CI)**	**Annual Cases***	**CIR**	**ASR (95% CI)**	**Annual Cases***	**CIR**	**ASR (95% CI)**
Incidence Overall												
Males	**54**	**1.57**	**1.44 (1.23–1.66)**	**59**	**1.66**	**1.42 (1.23–1.62)**	**57**	**1.49**	**1.23 (1.07–1.40)**	**61**	**1.48**	**1.23 (1.09–1.39)**
<60 years	27	0.95	0.90 (0.72–1.10)	24	0.83	0.74 (0.59–0.92)	22	0.72	0.66 (0.53–0.81)	29	0.91	0.81 (0.68–0.97)
60–69 years	7	2.35	2.36 (1.52–3.48)	10	2.87	2.87 (2.00–3.99)	11	2.62	2.62 (1.89–3.54)	12	2.79	2.77 (2.08–3.62)
70–79 years	12	5.84	5.90 (4.22–8.04)	15	6.92	6.91 (5.19–9.02)	14	5.62	5.48 (4.13–7.14)	12	4.18	4.20 (3.15–5.48)
80+ years	8	8.99	8.70 (5.75–12.64))	10	9.54	9.46 (6.59–13.17)	11	8.32	8.32 (5.98–11.29)	7	4.67	4.57 (3.09–6.51)
Females	**39**	**1.09**	**0.82 (0.68–0.99)**	**49**	**1.31**	**0.97 (0.82–1.14A**	**41**	**1.03**	**0.80 (0.68–0.94)**	**42**	**1.01**	**0.75 (0.64–0.88)**
<60 years	15	0.53	0.49 (0.36–0.64)	17	0.60	0.54 (0.41–0.69)	16	0.55	0.49 (0.37–0.62)	18	0.59	0.51 (0.41–0.64)
60–69 years	8	2.22	2.22 (1.46–3.23)	11	2.83	2.81 (1.99–3.86)	10	2.40	2.38 (1.71–3.23)	8	1.68	1.67 (1.15–2.33)
70–79 years	6	2.24	2.11 (1.32–3.21)	9	3.18	3.16 (2.18–4.42)	8	2.60	2.51 (1.71–3.56)	7	2.04	2.01 (1.36–2.86)
80+ years	10	5.48	5.49 (3.87–7.57)	12	5.45	5.46 (3.94–7.38)	6	2.60	2.59 (1.68–3.82)	9	3.47	3.48 (2.49–4.75)
Total	**93**	**1.33**	**1.10 (0.98–1.24)**	**108**	**1.48**	**1.17 (1.05–1.29)**	**98**	**1.26**	**0.99 (0.89–1.10)**	**103**	**1.24**	**0.99 (0.90–1.09)**

<60 years	42	0.74	0.69 (0.58–0.82)	41	0.72	0.64 (0.54–0.75)	38	0.64	0.57 (0.49–0.67)	48	0.75	0.67 (0.58–0.76)
60–69 years	15	2.28	2.28 (1.71–3.00)	20	2.85	2.84 (2.23–3.58)	21	2.51	2.50 (1.99–3.10)	20	2.22	2.21 (1.77–2.72)
70–79 years	18	3.72	3.70 (2.84–4.75)	25	4.76	4.75 (3.80–5.86)	22	3.94	3.81 (3.05–4.71)	19	3.02	3.03 (2.42–3.75)
80+ years	19	6.59	6.55 (5.05–8.35)	22	6.80	6.82 (5.39–8.52)	17	4.56	4.56 (3.54–5.79)	16	3.9	3.90 (3.04–4.92)

* Incidence: mean annual case frequency extrapolated to the whole Swiss population from cases observed in the cancer registries. Discrepancies between the sum and total number of expected cases are caused by rounding errors. CIR: crude rates per 100’000 py. ASR: age–standardized incidence–rates per 100,000 py (old European standard).

Table S2: Mortality of CML, 1995–2000, 2001–2006, 2007–2012, and 2013–2017.

Variation	1995–2000			2001–2006			2007–2012			2013–2017		
	**Annual Cases***	**CR**	**ASR (95% CI)**	**Annual Cases***	**CR**	**ASR (95% CI)**	**Annual Cases***	**CR**	**ASR (95% CI)**	**Annual Cases***	**CR**	**ASR (95% CI)**
Mortality Overall												
Males	**38**	**1.09**	**1.00 (0.87–1.14)**	**27**	**0.75**	**0.61 (0.52–0.71)**	**27**	**0.70**	**0.51 (0.44–0.60)**	**18**	**0.44**	**0.30 (0.24–0.37)**
<60 years	12	0.43	0.42 (0.33–0.52)	5	0.16	0.15 (0.10–0.21)	2	0.07	0.06 (0.03–0.11)	2	0.07	0.06 (0.03–0.11)
60–69 years	8	2.68	2.67 (1.97–3.53)	4	1.07	1.08 (0.67–1.63)	5	1.10	1.10 (0.73–1.60)	3	0.68	0.67 (0.37–1.10)
70–79 years	9	4.35	4.32 (3.2–5.67)	9	4.16	4.10 (3.09–5.34)	9	3.72	3.62 (2.73–4.72)	4	1.51	1.49 (0.93–2.26)
80+ years	9	9.59	9.75 (7.25–12.84)	9	8.74	9.32 (7.01–12.13)	11	8.61	8.99 (6.93–11.45)	8	5.44	5.58 (3.99–7.58)
Females	**34**	**0.93**	**0.64 (0.55–0.74)**	**25**	**0.67**	**0.42 (0.35–0.50)**	**16**	**0.40**	**0.21 (0.16–0.26)**	**13**	**0.31**	**0.13 (0.10–0.18)**
<60 years	8	0.28	0.26 (0.19–0.35)	4	0.13	0.12 (0.08–0.19)	1	0.02	0.02 (0.01–0.05)	1	0.03	0.02 (0.01–0.06)
60–69 years	6	1.83	1.80 (1.27–2.47)	5	1.26	1.25 (0.83–1.81)	3	0.62	0.62 (0.36–1.01)	1	0.22	0.21 (0.07–0.50)

70–79 years	8	2.67	2.59 (1.88–3.47)	7	2.18	2.08 (1.47–2.85)	4	1.35	1.30 (0.84–1.93)	2	0.52	0.49 (0.22–0.94)
80+ years	12	6.28	6.28 (4.91–7.91)	10	4.76	4.77 (3.65–6.13)	8	3.45	3.45 (2.56–4.55)	9	3.59	3.54 (2.60–4.71)
Total	**72**	**1.01**	**0.80 (0.72–0.88)**	**52**	**0.71**	**0.49 (0.43–0.55)**	**43**	**0.55**	**0.33 (0.29–0.38)**	**31**	**0.37**	**0.21 (0.17–0.25)**
<60 years	20	0.35	0.34 (0.28–0.41)	9	0.15	0.13 (0.10–0.18)	3	0.05	0.04 (0.02–0.07)	3	0.05	0.04 (0.02–0.07)
60–69 years	15	2.23	2.21 (1.77–2.72)	8	1.17	1.17 (0.87–1.54)	7	0.86	0.86 (0.62–1.15)	4	0.45	0.43 (0.27–0.67)
70–79 years	16	3.37	3.31 (2.68–4.04)	16	3.03	2.94 (2.37–3.60)	13	2.40	2.32 (1.84–2.89)	6	0.97	0.95 (0.64–1.35)
80+ years	21	7.34	7.38 (6.13–8.80)	20	6.09	6.19 (5.12–7.42)	19	5.24	5.27 (4.36–6.32)	18	4.26	4.28 (3.43–5.28)

* Mortality: mean annual case frequency derived from nationwide cause of death statistics. Discrepancies between the sum and total number of expected cases are caused by rounding errors. CR: crude rates of mortality per 100’000 py. ASR: age–standardized mortality–rates per 100,000 py (old European standard).

Table S3: OS and RS at 5 years of CML patients in the Swiss CCR.

Time Period	Age Group	Observed Survival (%)	Relative Survival (%)	95% CI Lower Limit	95% CI Upper Limit	Standard Error RS (%)
1995–2000	all ages	32.4	35.7	29.8	41.6	3.0
2001–2006	all ages	48.9	53.2	46.6	59.3	3.3
2007–2012	all ages	64.1	70.7	64.8	75.8	2.8
2013–2017	all ages	67.7	74.4	67.6	80.0	3.2
1995–2000	<60	55.3	56.1	46.0	65.2	4.9
2001–2006	<60	81.0	82.1	74.2	87.8	3.4
2007–2012	<60	92.8	93.8	88.6	96.9	2.0
2013–2017	<60	90.9	91.9	86.2	95.5	2.3
1995–2000	60–69	45.5	48.8	35.9	60.9	6.5
2001–2006	60–69	53.8	57.0	42.2	69.8	7.1
2007–2012	60–69	67.8	71.3	59.7	80.6	5.3
2013–2017	60–69	80.7	84.7	72.9	92.5	4.9
1995–2000	70–79	19.7	24.2	14.1	36.3	5.8
2001–2006	70–79	40.8	48.9	34.7	62.7	7.3
2007–2012	70–79	54.3	63.6	51.0	74.7	6.1
2013–2017	70–79	50.8	58.3	43.9	71.2	7.0
1995–2000	80+	3.5	6.8	1.4	19.4	4.4
2001–2006	80+	5.2	8.9	2.5	21.6	4.8
2007–2012	80+	24.3	42.6	23.8	64.2	10.6
2013–2017	80+	30.2	52.8	32.3	74.9	11.1

Table S4. Significance of 5yrs RS of CML patients in the Swiss CCR.

All Ages	z	*p*–Value	<60 years	z	*p*–Value	60–69 Years	z	*p*–Value	70–79 Years		*p*–Value	80+ Years	z	*p*–Value
t1 vs. t2	3.9335	<0.001	t1 vs. t2	4.3226	<0.001	t1 vs. t2	0.8486	0.396	t1 vs. t2	2.6677	0.008	t1 vs. t2	0.3232	0.747
t2 vs. t3	4.0669	<0.001	t2 vs. t3	2.9292	0.003	t2 vs. t3	1.6123	0.107	t2 vs. t3	1.5395	0.124	t2 vs. t3	2.9075	0.004
t3 vs. t4	0.8807	0.378	t3 vs. t4	0.6209	0.535	t3 vs. t4	1.8401	0.066	t3 vs. t4	0.5611	0.575	t3 vs. t4	0.6716	0.502

Table S5. Summary of other population–based CML studies.

Study	Registry/Database	Observation Time	No. of CML pts.	Relative Survival–5 Years					
[1]Daskalakis et al., Swiss NICER CML study group [1]	CH, NACR	1995–2014	1236				0.35 (1995–2000)	0.52 (2001–2006)	0.68 (2007–2014)
[2]Chen Y et al., Leuk Lymphoma 2013 July [2]	US, SEER–9–registries database	1975–2009	11888	0.26 (1975–1989)			0.36 (1990–2000)	0.56 (2001–2009)	
[3]Roman E et al., Cancer Epidemiol 2016 Jun [3]	UK, Haematological Malignancy Research Network	2004–2013	318						0.90 (2004–2015)
[4]Smith AG et al., BMJ Open 2014 Jan [4]	UK, Haematological Malignancy Research Network	2004–2011	242					0.89 (2004–2011)	
[5]Beinortas T et al., BMC Cancer 2016 Mar [5]	Lithuania, National Haematological Disease Monitoring System	2000–2013	601				0.33 (2000–2004)	0.55 (2005–2009)	NR (2010–2013)
[6]Thielen N et al., Europ J Haematol 2016 Aug [6]	Netherland Cancer Registry	1989–2012	3585			0.36 (1989–2000)		0.69 (2001–2006)	0.79 (2007–2012)
[7]Penot A et al., Leuk Lymphoma 2015 Jun [7]	France, 5 Cancer Registries	1980–2009	781		0.44 (1980–1986)		0.64 (1987–1999)	0.89 (2000–2009)	
[8]Corm S et al., Blood 2011 Okt [8]	France, CML–Registry of Nord–Pas de Calais	1990–2007	691			0.64 (1990–1994)	0.66 (1995–1999)	0.88 (2000–2007)	
[9]Björkholm M et al., JCO June 2011 [9]	Swedish Cancer Registry	1973–2008	3173	0.21 (1973–1979)	0.23 (1980–1986)	0.37 (1987–1993)	0.54 (1994–2000)	0.80 (2001–2008)	
[10]Gunnarsson N et al., Europ J Haematol 2016 Oct [10]	Swedish Cancer Registry	1970–2012	1137	0.18 (1970)					0.82 (2012)

[1] RS was estimated using the Ederer–II–method and the period approach. The estimates are age–standardized according to ICSS Standard 1. [2] RS survival was estimated using the Hakulinen–method and the cohort approach. [3] RS survival was estimated using the model–based method of Estéve 1990. [4] RS survival was estimated using the model–based method of Estéve 1990. [5] RS survival was estimated using the Ederer–II–method and the cohort approach. [6] RS was estimated using a cohort approach (estimation method unknown). [7] RS was estimated using a cohort approach (estimation method unknown).[8] RS was estimated using flexible parametric regression models. [9] RS was estimated using the Ederer–II–method and the cohort approach.10 RS was estimated using the period approach. The RS estimates for the years between 1970 and 2012 were only presented graphically.

References:

1. Daskalakis, M.; Feller, A.; Noetzli, J.; Bonadies, N.; Arndt, V.; Baerlocher, G.M.; the NICER Working Group. Potential to Improve Therapy of Chronic Myeloid Leukemia (CML), Especially for Patients with Older Age: Incidence, Mortality, and Survival Rates of Patients with CML in Switzerland from 1995–2017. *Cancers 2021, 13, x.* https://doi.org/10.3390/xxxxx
2. Chen, Y.; Wang, H.; Kantarjian, H.; Cortes, J. Trends in chronic myeloid leukemia incidence and survival in the United States from 1975 to 2009. *Leukemia & lymphoma* **2013**, *54*, 1411–1417, doi:10.3109/10428194.2012.745525.
3. Roman, E.; Smith, A.; Appleton, S.; Crouch, S.; Kelly, R.; Kinsey, S.; Cargo, C.; Patmore, R. Myeloid malignancies in the real-world: Occurrence, progression and survival in the UK's population-based Haematological Malignancy Research Network 2004-15. *Cancer epidemiology* **2016**, *42*, 186–198, doi:10.1016/j.canep.2016.03.011.
4. Smith, A.G.; Painter, D.; Howell, D.A.; Evans, P.; Smith, G.; Patmore, R.; Jack, A.; Roman, E. Determinants of survival in patients with chronic myeloid leukaemia treated in the new era of oral therapy: findings from a UK population-based patient cohort. *BMJ open* **2014**, *4*, e004266, doi:10.1136/bmjopen-2013-004266.
5. Beinortas, T.; Tavoriene, I.; Zvirblis, T.; Gerbutavicius, R.; Jurgutis, M.; Griskevicius, L. Chronic myeloid leukemia incidence, survival and accessibility of tyrosine kinase inhibitors: a report from population-based Lithuanian haematological disease registry 2000-2013. *BMC cancer* **2016**, 16, 198, doi:10.1186/s12885-016-2238-9.
6. Thielen, N.; Visser, O.; Ossenkoppele, G.; Janssen, J. Chronic myeloid leukemia in the Netherlands: a population-based study on incidence, treatment, and survival in 3585 patients from 1989 to 2012. *European journal of haematology* **2016**, *97*, 145–154, doi:10.1111/ejh.12695.
7. Penot, A.; Preux, P.M.; Le Guyader, S.; Collignon, A.; Herry, A.; Dufour, V.; Monnereau, A.; Woronoff, A.S.; Troussard, X.; Pons, E.; et al. Incidence of chronic myeloid leukemia and patient survival: results of five French population-based cancer registries 1980-2009. *Leukemia & lymphoma* **2015**, *56*, 1771–1777, doi:10.3109/10428194.2014.974046.
8. Corm, S.; Roche, L.; Micol, J.B.; Coiteux, V.; Bossard, N.; Nicolini, F.E.; Iwaz, J.; Preudhomme, C.; Roche-Lestienne, C.; Facon, T.; et al. Changes in the dynamics of the excess mortality rate in chronic phase-chronic myeloid leukemia over 1990-2007: a population study. *Blood* **2011**, *118*, 4331-4337, doi:10.1182/blood-2011-01-330332.
9. Bjorkholm, M.; Ohm, L.; Eloranta, S.; Derolf, A.; Hultcrantz, M.; Sjoberg, J.; Andersson, T.; Hoglund, M.; Richter, J.; Landgren, O.; et al. Success story of targeted therapy in chronic myeloid leukemia: a population-based study of patients diagnosed in Sweden from 1973 to 2008. *Journal of clinical oncology : official journal of the American Society of Clinical Oncology* **2011**, *29*, 2514-2520, doi:10.1200/jco.2011.34.7146.
10. Gunnarsson, N.; Sandin, F.; Hoglund, M.; Stenke, L.; Bjorkholm, M.; Lambe, M.; Olsson-Stromberg, U.; Richter, J.; Sjalander, A. Population-based assessment of chronic myeloid leukemia in Sweden: striking increase in survival and prevalence. *European journal of haematology* **2016**, *97*, 387-392, doi:10.1111/ejh.12743.
